# Long non-coding RNA and circular RNA and coding RNA profiling of plasma exosomes of osteosarcoma by RNA seq

**DOI:** 10.1038/s41597-023-02295-9

**Published:** 2023-06-22

**Authors:** Yun Liu, Haijun Tang, Chong Li, Nenggan Huang, Jifeng Miao, Lin Chen, Kai Luo, Feicui Li, Shangyu Liu, Shijie Liao, Wenyu Feng, Xinli Zhan, Tianyu Xie, Wei Tang, Qingjun Wei, Jili Lu

**Affiliations:** 1 Department of Spine and Osteopathic Surgery, The First Affiliated Hospital of Guangxi Medical University, Nanning, Guangxi China; 2 Department of Joint surgery, Baise People’s Hospital, Baise, Guangxi China; 3 Department of Joint surgery, Affiliated Southwest Hospital of Youjiang Medical University for Nationalities, Baise, Guangxi China

**Keywords:** Bone cancer, Cancer screening

## Abstract

Osteosarcoma (OS) is a primary bone tumor with high malignancy and the mechanism of hematogenous metastasis in OS is still not clear. The plasma exosomes derived from osteosarcoma play a key role in the process of tumor metastasis. Here, we established RNA-seq dataset for lncRNAs, circRNAs and mRNAs in plasma exosomes from 10 OS patients and 5 healthy donors. A total of 329.52 Gb of clean data was obtained. Besides, 1754 lincRNAs, 7096 known and 1935 new circRNA was identified. Finally, gene expression profiles and differentially expressed genes (DEGs) were analyzed among these 15 samples. There were 331 DEGs of mRNA, 132 of lincRNA and 489 of circRNA was obtained, respectively. This data set provides a significant resource for relevant researchers to excavate potential dysregulated lncRNAs, circRNAs and mRNAs of plasma exosomes in OS versus normal conditions.

## Background & Summary

Osteosarcoma (OS), which mainly occur in children and adolescents, is a primary bone tumor with high malignancy^1^. Despite great efforts have made in the past 20 years, it is still difficult to exceed the survival rate of OS to 60%. The main reason for this poor prognosis is that OS is prone to lung metastasis via hematogenous channel^2^. However, the mechanism of hematogenous metastasis in OS is still not clear.

Exosomes, a tiny extracellular vesicle with a diameter of 40–100 nm, can be released by various cells^3^. Exosomes contains many substances, such as long non-coding RNAs (lncRNAs), microRNAs (miRNA), circular RNAs (circRNAs) and protein. Thus, exosomes are regarded as the medium of intercellular communication, which are closely related to the many kinds of diseases^4^. It should be noted that tumor-derived exosomes can enter into blood and lead to tumor metastasis. Researches show that the plasma exosomes derived from osteosarcoma play a key role in the process of tumor metastasis^5^. However, the specific biological function of plasma exosomes to cancer metastasis remains unclear.

LncRNAs present linear chain structure with transcripts ≥200 nucleotides in length, while circRNAs is an enclosed circle structure without 3′-end and 5′-end. Although both lncRNAs and circRNAs belong to non-coding RNAs, they make a critical difference to regulation of mRNA^6^. LncRNAs, circRNAs and mRNAs are the main small molecular substance in exosomes, playing a crucial role in biological function of exosomes. A growing number of evidence show that exosomes secreted by primary tumor cells can carry these three types of molecules to the metastatic lesions and regulate the biological behavior of metastatic cells^7^. Thus, to make a profound study on these three types of RNA in plasma exosomes is essential to control cancer metastasis. RNA-seq is an effective and comprehensive method to explore RNA expression in exosomes. Data set of RNA seq of blood exosomes in various disease has been established^8^. However, transcriptome profiling of plasma exosomes in OS has not been systematically explored.

Here, we established RNA-seq dataset for lncRNAs, circRNAs and mRNAs in plasma exosomes from 10 OS patients and 5 healthy donors. Although miRNA is not captured in the present study for the small sample and technical defects, this data set still provides a significant resource for relevant researchers to excavate potential dysregulated lncRNAs, circRNAs and mRNAs of plasma exosomes in OS versus normal conditions.

## Methods and Result

### Ethical approval

This study was supported by the Ethics Committee of the First Affiliated Hospital of Guangxi Medical University (2019(KY-E-162)). All steps are carried out according to the standards of the Ethics Committee. Patients and healthy volunteers in this research were informed of the study, including statement that their sequencing data would be shared. Both patients and healthy volunteers signed the informed consent.

### Subjects and sample collection

**Fig. 1:**
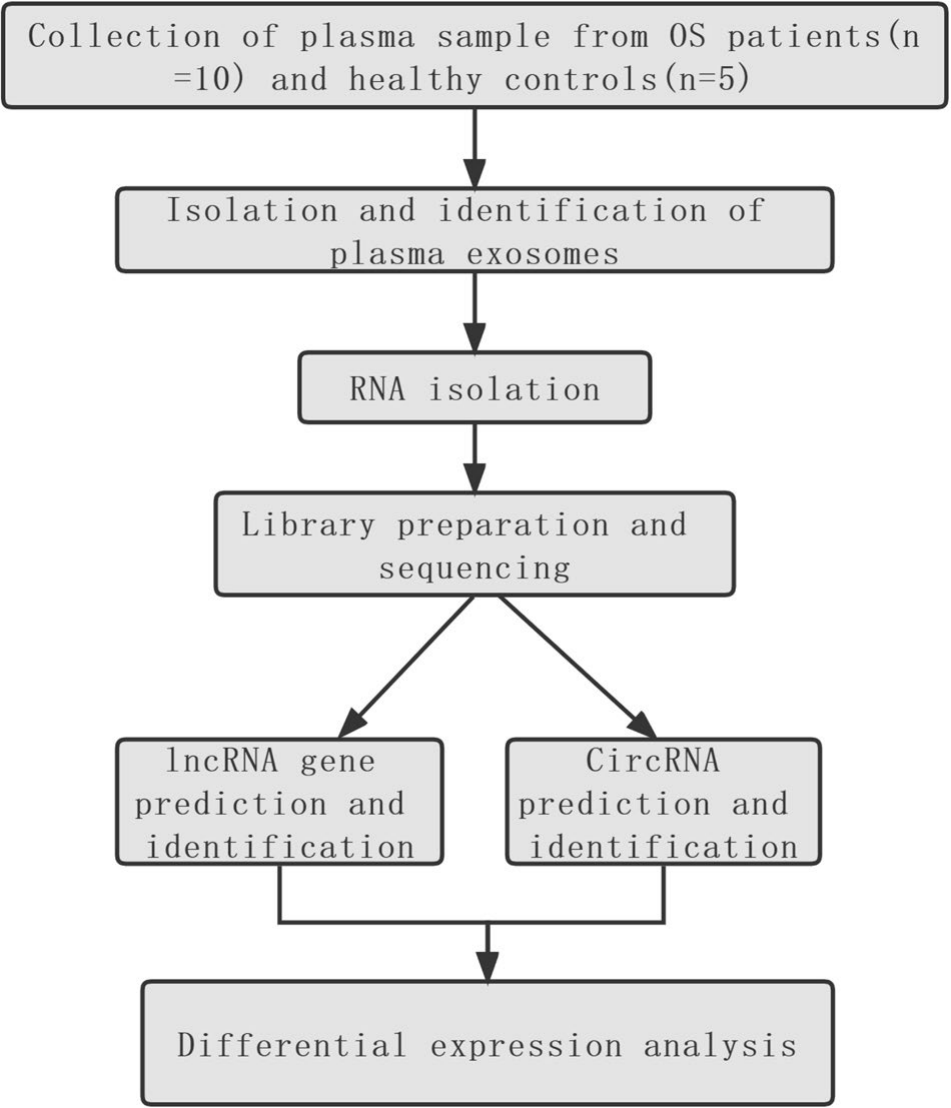
The experimental design and all workflow of this study.

The experimental design and all workflow were presented in Fig. 1. To make the results more comparable, we developed elaborate inclusion and exclusion criteria for both patients and controls. Inclusion criteria: 1) patients were diagnosed with OS by postoperative pathological examination of resected specimens. 2) patients with tumor located in the extremities. 3) patients with initial treatment. Exclusion criteria: 1) patients and controls with infectious diseases, such as fever, hepatitis B infection, HIV, autoimmune disease and etc. 2) patients and controls with hemopathy, endocrine disease, and so on. 10 OS patients (6 female and 4 male) and 5 age- and sex-matched healthy controls were ultimately included in this study. According to surgical grading of bone tumor, 2 patients were classified into III, 5 patients into IIB, 2 patients into IB, and 1 patient into IIA, respectively (Table 1). 10 ml peripheral blood was collected from patients and controls in the morning with an empty stomach. Peripheral blood samples will be centrifuged at 300 min for 15 min to obtain blood plasma.

**Table 1:** General Information of patients and volunteers.

Patient	GEO	Group	Sex	Age (years)	Location	Grade
A503-02	GSM6751275	osteosarcoma	Female	23	Proximal tibia	IIB
A503-03	GSM6751276	osteosarcoma	Male	5	Distal femur	IIB
A503-04	GSM6751277	osteosarcoma	Female	15	Proximal femur	IIB
A503-05	GSM6751278	osteosarcoma	Female	20	Proximal femur	IIA
A503-06	GSM6751279	osteosarcoma	Female	15	Proximal tibia	IIB
AA230-001	GSM7156629	osteosarcoma	Male	16	Distal femur	III
AA230-002	GSM7156630	osteosarcoma	Female	11	Proximal fibula	IB
AA230-003	GSM7156631	osteosarcoma	Male	14	Distal femur	III
AA230-004	GSM7156632	osteosarcoma	Male	12	Distal femur	IIB
AA230-005	GSM7156633	osteosarcoma	Female	18	Distal femur	IB
A503-07	GSM6751280	control	Female	20	NA	NA
A503-08	GSM6751281	control	Male	8	NA	NA
A503-09	GSM6751282	control	Female	17	NA	NA
A503-10	GSM6751283	control	Female	20	NA	NA
A503-11	GSM6751284	control	Female	18	NA	NA

### Isolation of plasma exosomes

Firstly, plasma sample from individuals would be underwent a procedure of ultracentrifuged, which is similar to previous reports^9^. 7-fold phosphate-buffered saline (PBS) was added to the plasma sample to dilute the sample, centrifuged under condition of 13,000 × g for 30 minutes, and removed large particles via a 0.22 μm filter. The above supernatant would be centrifuged again for 2 hours at at 100,000 × g, 4 °C and then the exosomes pellet was obtained. The pellet was re-suspended with PBS and centrifuged again at 100,000 × g 4 °C for 2 h. Finally, 100 µl PBS were put into pellet to re-suspende the exosomes.

### Identification of exosomes

The isolated exosomes should be identified at three levels: nanoparticle tracking analysis (NTA), transmission electron microscopy (TEM), and western blot analysis (WB).

#### Nanoparticle tracking analysis (NTA)

**Fig. 2:**
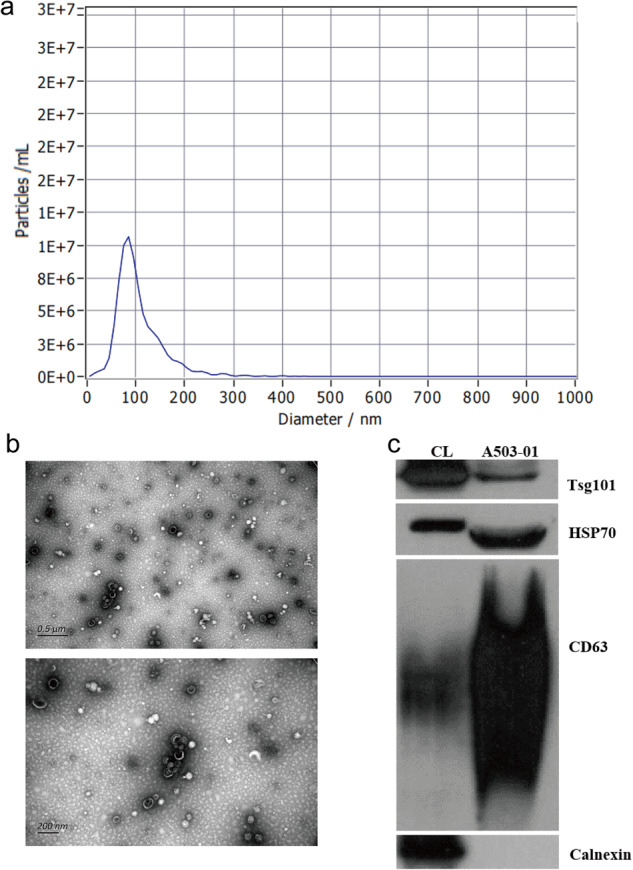
Identification of exosomes. (**a**) The result of Nanoparticle tracking analysis (NTA) showed that the mean diameter of exosomes was 105.3 nm and the Wave Crest of diameter was located in 85.2 nm. (**b**) Transmission electron microscopy (TEM) showed that cup sharp of exosomes were clearly visible. (**c**) Western blot analysis (WB) demonstrated that compared to positive control group (CL), positive protein (CD63, HSP70, TSG101) was high expressed in our identified exosomes, while negative protein (calnexin) didn’t express.

We used ZetaView PMX 110 (Particle Metrix, Meerbusch, Germany) equipped with 405 nm laser to determine the size and quantity of isolated particles. A video with a duration of 60 seconds was shot at a frame rate of 30 frames per second, and particle motion was analyzed using NTA software (ZetaView 8.02.28). Our result showed the mean diameter of exosomes was 105.3 nm and the main peak of diameter was located in 85.2 nm with a proportion 98.4%. Besides, the concentration was 1.5E + 12 particles/ mL (Fig. 2a and Table 2).

**Table 2:** Detection result of Nanoparticle tracking analysis (NTA).

Volume (μl)	200
Average diameter (nm)	105.3
Main peak (nm)	85.2
Percentage of main peak (%)	98.4
Concentration (Particles/mL)	1.50E + 12

#### Transmission electron microscopy (TEM)

10 µl exosomes sample of was placed on the copper mesh of electron microscope at room temperature for 1 min. After absorbing the floating liquid with filter paper, 15 µl of 2% uranyl acetate solution was took to stain for 1 min. The sample was then moved to an incandescent lamp and grilled for 2 minutes. The copper mesh was observed and photographed under a TME (H-7650, Hitachi Ltd., Tokyo, Japan). The morphology of exosomes in this study was observed in Fig. 2b.

#### Western blot analysis (WB)

WB was used to detect the exosomes marker proteins CD63, HSP90, TSG101 and calnexin. The sample stock solution was diluted according to the detected protein concentration. The sample was transferred to PVDF membrane after SDS-PAGE electrophoresis. Rabbit polyclonal antibody of above exosomes marker were added to the samples, and then incubated at 4 °C for 1 hour. Finally, IgG secondary antibody (all diluted according to 1:5000) and enhanced chemotherapy (ECL) was added to develop images. The result of WB analysis demonstrated that compared to positive control group (CL), positive protein (CD63, HSP70, TSG101) was high expressed in our identified exosomes, while negative protein (calnexin) didn’t express (Fig. 2c).

### ExoRNA isolation and RNA analyses

MiRNeasy Plasma Advanced Kit (Qiagen, cat. No. 217204), which can purify total RNA from a small amount of plasma, was used in this study. Briefly, dissolve the serum sample in QIAzol lysis reagent, add chloroform, and centrifuge. Take the upper aqueous phase, add ethanol and RNeasy Minelute centrifuge column, and the total RNA will be bound to the membrane. Then we used Agilent Bioanalyzer 2100 System (Agilent Technologies, CA, USA) to perform the quality control of RNA sample.

### Library preparation and sequencing

Ribosomal RNA accounts for a high proportion (~90%) of total RNA, which need to be removed before library preparation. SMARTer Stranded Total RNA Seq Kit V2 (Takara Bio USA, Inc.), which is designed to efficiently prepare Illumina sequencing library from picogram level total RNA (250 pg-10 ng), was used in this study according to the manufacturer’s instructions. The evaluation of library quality was performed on the Agilent Bioanalyzer 2100 and Quantitative real-time PCR. The clustering of the index-coded samples was performed on acBot Cluster Generation System using TruSeq PE Cluster Kitv3-cBot-HS (Illumina, San Diego, CA, USA). The resulting library will then be sequenced via an Illumina Hiseq platform and paired-end reads were generated.

### Quality validation of the raw data and library

Clean data (clean reads) were obtained by removing reads containing adapter, reads containing ploy-N and low-quality reads from raw data using Cutadapt software of version 2.10 (https://cutadapt.readthedocs.io/en/stable/). At the same time, Q20, Q30, GC-content and sequence duplication level of the clean data were calculated. The percentage of Q30 of each sample was not less than 88.74% in the present study, which released that the raw data in the present study was accredited (Table 3). After quality control of raw data, 329.52 Gb clean data was obtained.

**Table 3:** RNA-seq read statistics.

Sample	Clean Reads	GC%	Q30%	Mapped Reads	Mapped Reads%	Uniq Map Reads	Uniq Map Reads%
A503-02	162,933,364	59.26	90.4	157,326,706	96.56	96,220,319	59.06
A503-03	125,969,246	56.04	92.72	123,328,224	97.90	90,088,508	71.52
A503-04	135,632,620	58.58	92.72	130,501,638	96.22	82,482,421	60.81
A503-05	132,616,972	57.47	92.52	128,503,400	96.90	84,491,103	63.71
A503-06	133,739,188	56.41	92.9	127,906,602	95.64	83,795,108	62.66
AA230-001	160,447,526	63.58	90.07	160,054,878	99.76	75,565,972	47.10
AA230-002	180,283,724	64.61	89.07	178,679,940	99.11	76,598,452	42.49
AA230-003	181,390,626	62.08	89.83	179,186,168	98.78	88,953,344	49.04
AA230-004	143,039,052	63.40	90.09	141,549,190	98.96	64,818,658	45.32
AA230-005	165,885,740	64.59	88.74	165,101,130	99.53	69,979,229	42.19
A503-07	196,008,702	60.27	90.61	188,046,266	95.94	107,389,451	54.79
A503-08	171,314,664	56.51	90.43	162,308,422	94.74	103,626,831	60.49
A503-09	138,417,066	61.37	91.54	135,326,766	97.77	78,476,397	56.70
A503-10	122,826,034	60.34	91.86	120,510,080	98.11	70,596,251	57.48
A503-11	152,025,304	59.27	91.21	145,903,902	95.97	87,943,768	57.85

**Fig. 3:**
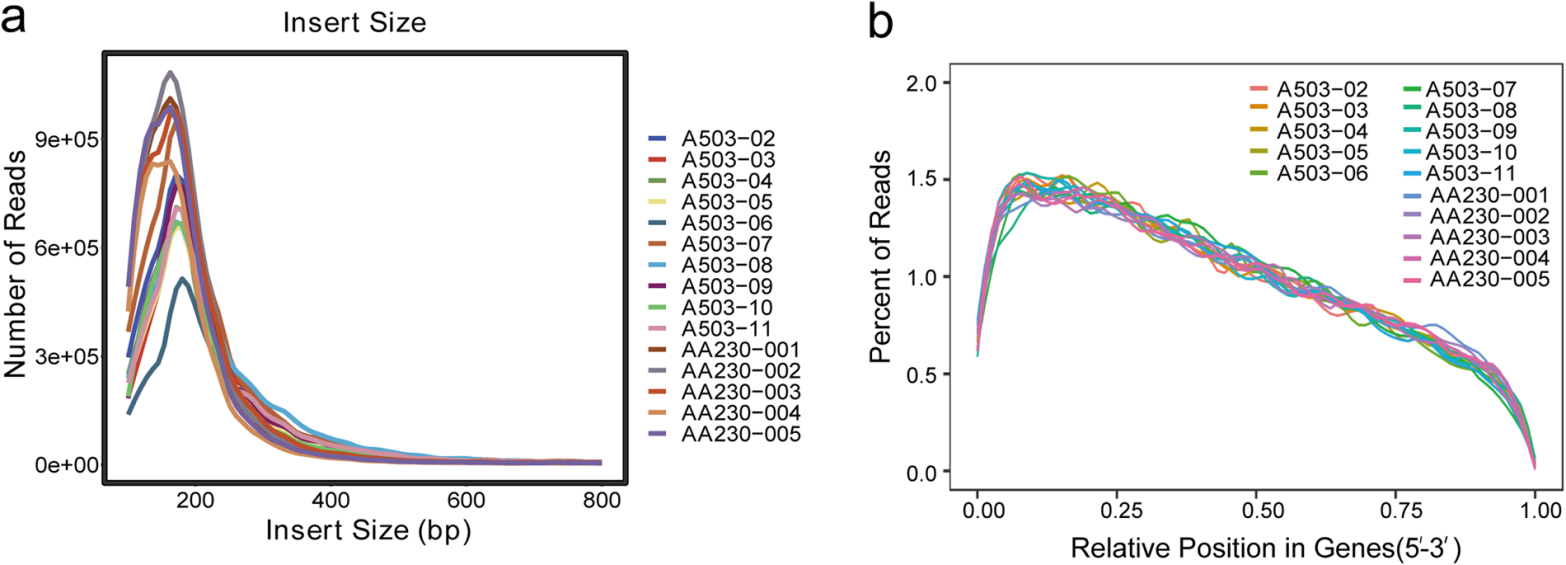
Quality validation of library. (**a**) Dispersion degree of estimated insert size. The result showed that the main peak of the curve falls near 200 bp. (**b**) Randomness distribution map. The result showed that a 3′ bias was present, which is due to the discontinuous nature of RNA of exosomes, but not related to mRNA degradation.

**Fig. 4:**
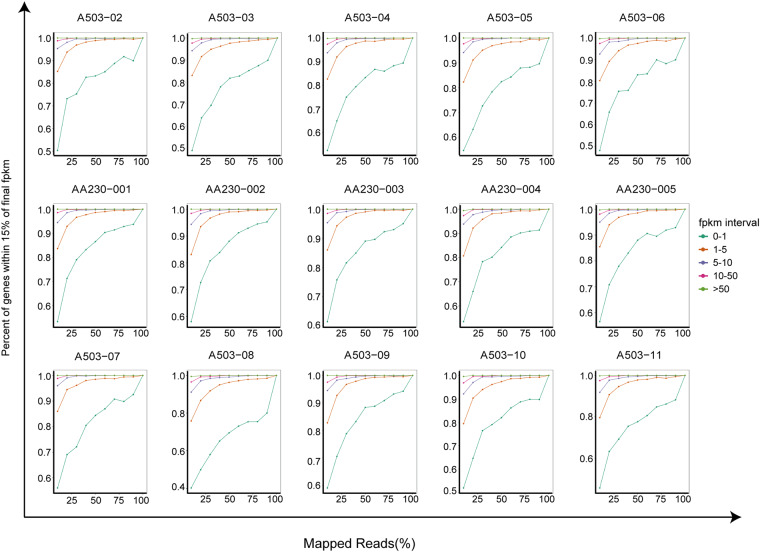
The saturation testing of the library. The result demonstrated that the number of genes detected in each sample is saturated, indicating that the sequencing depth of this study is sufficient.

In order to evaluate the quality of the library, we firstly estimate dispersion degree of estimated insert size. As showed in Fig. 3a, the main peak of the curve falls near 200 bp and does not deviate from the target area, indicating a small degree of dispersion in the length of the inserted fragment, and the selection of the inserted fragment size is normal. Subsequently, randomness distribution map was plotted to understand the degradation of mRNA. As we can see in Fig. 3b, a 3′ bias was found. This is due to the discontinuous nature of RNA of exosomes, but not related to mRNA degradation. Finally, to evaluate whether the data is sufficient and meet subsequent analysis, saturation testing is performed on the gene numbers. The result demonstrated that the number of genes detected in each sample is saturated, indicating that the sequencing depth of this study is sufficient (Fig. 4).

### Read alignment and transcript assembly

We used the designated genome GRCh38 (ftp://ftp.ensembl.org/pub/release-101/fasta/homo_sapiens/) as a reference for sequence alignment and subsequent analysis. For lncRNAs and mRNAs, we used the HISAT2 software (version 2.2.1.0)^10^ to align the reads and StringTie software (version 2.1.3)^11^ to assemble the aligned reads. Considering the feature of circRNA sequencing data, we used Burrows-Wheeler-Alignment Tool (https://sourceforge.net/ projects/bio-bwa/files/) to complete sequence alignment. The read mapping results of lncRNA, mRNA and circRNA were summarized in Table 3.

### lncRNA gene prediction and identification

**Fig. 5:**
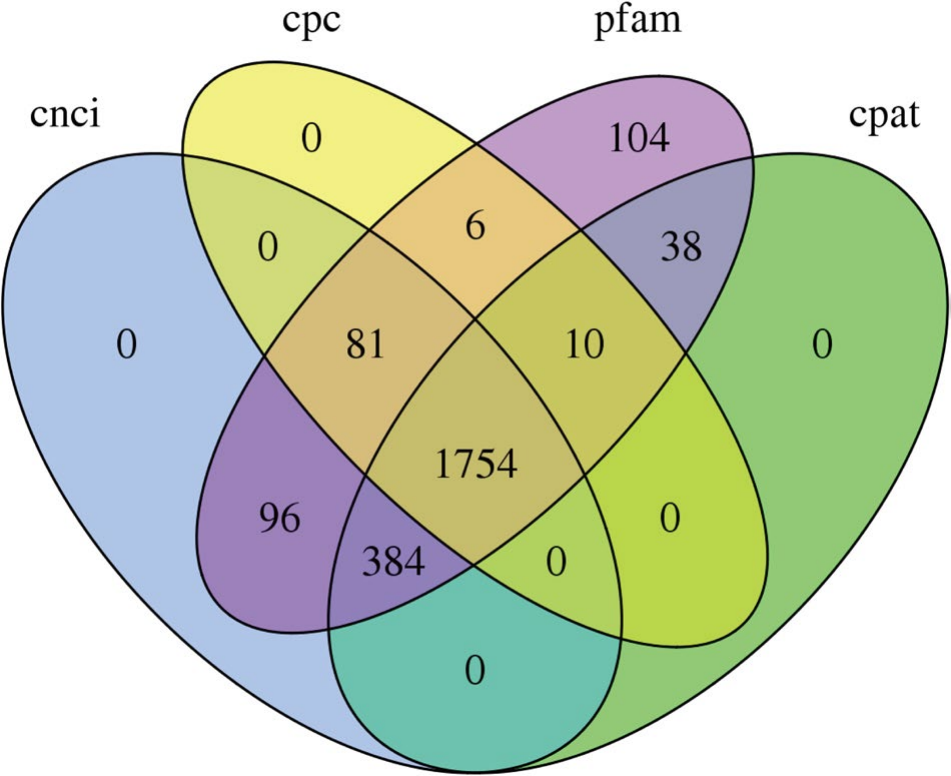
Venn diagrams of lncRNA gene prediction and identification by CPC, CNCI, Pfam and CPAT.

Cuffcompare program from the Cufflinks package v2.2.1 was firstly applied to compare the assembled transcripts with the existing gene annotation. We compared the results of assembled transcripts with the known lncRNA, and removed the known transcripts (class_code = c). The unknown transcripts were used to screen for putative lncRNAs. Four computational software for coding capability prediction, including CPC^12^, CNCI^13^, Pfam^14^ and CPAT^15^, were combined to sort non-protein coding RNA candidates from putative protein-coding RNAs in the unknown transcripts. The candidate lncRNA should meet the following conditions: (1)Transcription length ≥ 200 nt, (2) having more than two exons, (3) FPKM ≥ 0.1, 4) and class_code were “i”,“x”,“u”,“o”,“e”. Menwhile, further screened using above four computational approaches that have the power to distinguish the protein-coding genes from the non-coding genes. As well as the different types of lncRNAs include lincRNA, intronic lncRNA, anti-sense lncRNA were selected using cuff compare. The intersection of prediction results of several tools was taken as the prediction result of new lncRNAs. We ultimately obtained 1754 lincRNAs (Fig. 5). Supplementary Table 1 showed the prediction of the names of the lincRNA.

### CircRNA prediction and identification

Two algorithms of CIRI2 and find_circ were used to predict circRNA. In CIRI2^16^, by utilizing BWA software (Version: 7.12-r1039), the clean data was aligned to the reference gene set GRCh38 and the SAM files could be obtained. These SAM files were analyzed by CIRI2 software v2.0.5 (https://sourceforge.net/projects/ciri/files/CIRI2/) and ultimately gained the result of RNA prediction. Find_circ software (https://github.com/marvin- jens/find_circ/archive /v1.2.tar.gz) used bowtie2 (version 2.2.3) to complete the procedure of aligning and obtained predictive circRNA. The intersection gene obtained by the two algorithms will be used as the final circRNA. The predictive circRNA would be aligned to circBase database (http://www.circbase.org/) to identify known and new circRNA. We ultimately dentified 7096 known and 1935 new circRNA. Supplementary Tables 2, 3 showed the prediction of the known and new names of the circRNA, respectively.

### Expression abundance quantification of mRNA and lincRNA

Stringtie (https://ccb.jhu.edu/software/stringtie/index.shtml#pub) was applied to evaluate FPKMs of all mRNA and lncRNA genes in each sample. Gene FPKMs was computed by summing the FPKMs of transcripts in each gene group. FPKM means fragments per kilo-base of exon per million fragments mapped, calculated based on the length of the fragments and reads count mapped to this fragment. The formula of FPKMs: FPKM = cDNA fragments/Mapped fragments (Millions)*Transcript Length(kb).

### Expression abundance quantification of circRNA

Junction reads were used as circRNA expression level. We used TPM to normalize the expression abundance quantification. The formula of TPMs: TPMi = (Ni/Li)*1000000/sum(Ni/Li+………+ Nm/Lm). Ni is the junction reads of gene i and Li present exon length of gene i.

### Differential expression analysis of mRNA, lincRNA and circRNA

**Fig. 6:**
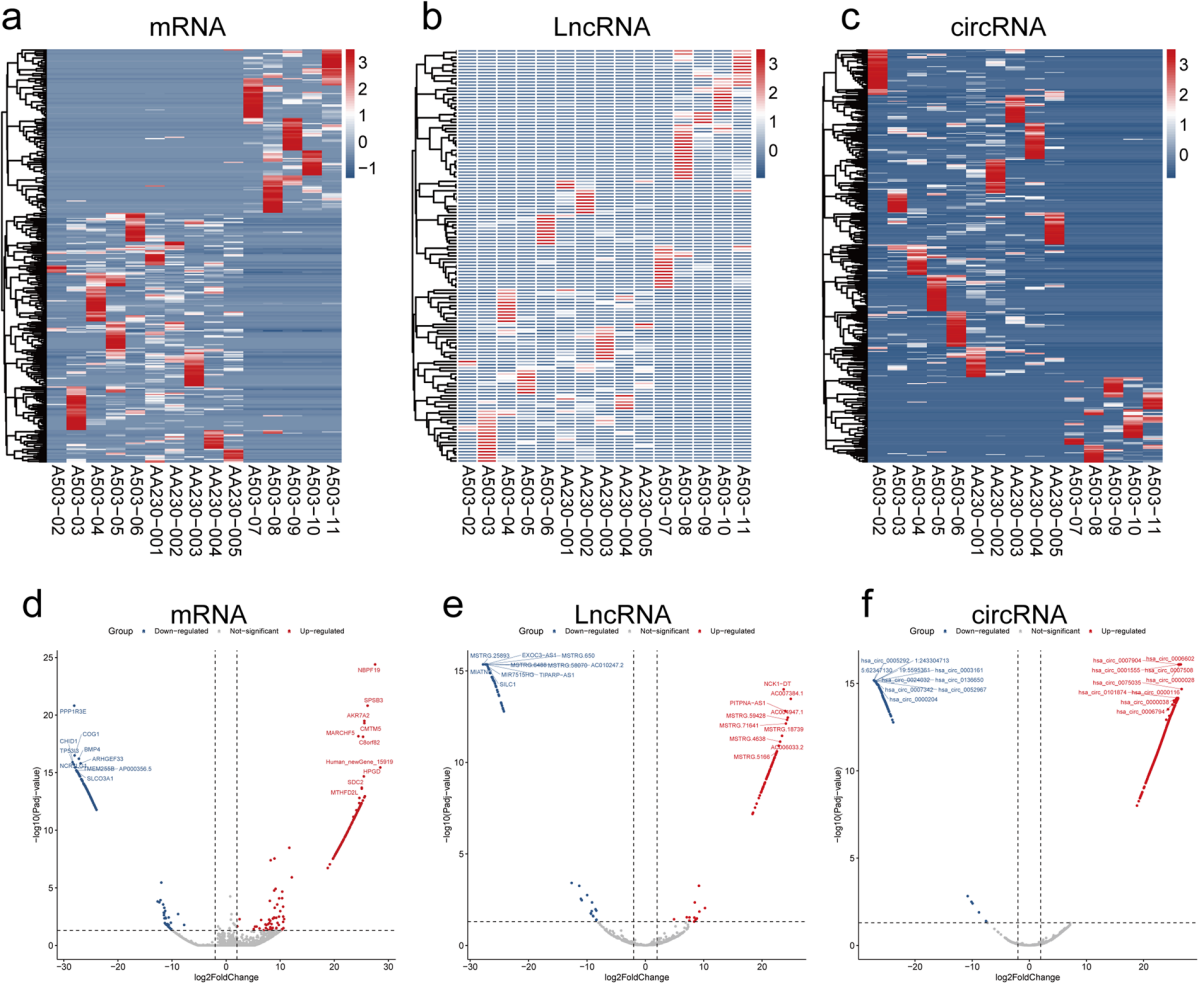
Heatmap of differentially expressed genes (DEGs) between OS patients and normal samples. (**a**) mRNA, (**b**) lincRNA, (**c**) circRNA. The DEGs and the name of top 20 DEGs of mRNA (**d**), lincRNA (**e**) and circRNA (**f**) were showed by the volcano plot.

The determination of differentially expressed genes (DEGs) between OS patients and normal samples was assessed by Deseq 2^17^. The screening criteria were |log2FC| ≥ 2 and pvalue < 0.05. Finally, there were 331 DEGs of mRNA (Fig. 6a and Supplementary Table 4), 132 of lincRNA (Fig. 6b and Supplementary Table 5) and 489 of circRNA (Fig. 6c and Supplementary Table 6), respectively. Besides, the DEGs and the name of top 20 DEGs of mRNA, lincRNA and circRNA were also showed in the volcano plot (Fig. 6d–f).

## Data Records

The FASTQ files for the raw data and BAM files have been deposited in NCBI Sequence Read Archive (SRA) under BioProject accession of PRJNA904243 and SRP409185^18^. The transcript abundance was deposited in the NCBI Gene Expression Omnibus (GEO) and the accession number was GSE218526^19^. The GEO accession number of each sample was listed in Table 1. The files of RNA prediction, expression abundance quantification and DEGs analysis was also deposited in figshare^20^.

## Technical Validation

### RNA integrity assessment

NanoDrop spectrophotometer was utilize to quickly determine the OD value of RNA samples at 260 and 280 nm. The ratio of A260/A280 range 1.8–2.0 was considered acceptable, otherwise it would be resampled. Subsequently, Agilent 2100 Bioanalyzer was used to evaluate RNA integrity.

### Quality validation of RNA data

In order to obtain clean data, Cutadapt software of version 2.10 (https://cutadapt.readthedocs.io/en/stable/) was first used to remove reads containing adapter. At the same time, Q20, Q30, GC-content and sequence duplication level of the clean data were calculated. Low quality read that met the following conditions would be excluded: 1) The reads in which the number of undetermined base pairs was greater than 10%; 2) Q30 < 88.74% .

**Table 4:** The name and the links of all database depositories.

Depository	Links to Depository
SRA	https://identifiers.org/ncbi/insdc.sra:SRP409185
GEO	https://identifers.org/geo:GSE218526
Figshare	10.6084/m9.figshare.22659328.v1
Github	https://github.com/tangaode/Plasma-exosomes

## Supplementary information

Table S2

Table S3

Table S4

Table S5

Table S6

Table S1

## Data Availability

Most steps were completed base on the public-domain software, except for the calculation of differential genes. All analytical code of DEGs is available on the GitHub repository (https://github.com/tangaode/Plasma-exosomes). The provided R code was run and tested using R 4.1.0. The name and the links of all database depositories was showed in Table 4.
